# Medical Feuds

**Published:** 1852-10

**Authors:** 


### MEDICAL FEUDS.

The Boston Medical and Surgical Journal refers thus to a quarrel now in progress between the Homœopathists and the Hydropathists:

By an article on hydropathy, in the Philadelphia Journal of Homœopathy, it seems that the infinitesimal gentlemen have no confidence in the professors of the water system of medication. They express a proper degree of horror for anything so shockingly unscientific as water, as a remedial agent. Now both schools utterly condemn and abominate the regular practice; and it is a little singular they should thus quarrel among themselves.

